# Supplementary material for: Effects of environmental conditions and catch processing on survival probability of plaice discards in the North Sea pulse trawl fishery
Source: PLoS One. 2023 Jun 14;18(6):e0287020. doi: 10.1371/journal.pone.0287020 (PMC10266594; doi:10.1371/journal.pone.0287020)
Supplement: Attachment — Submitted filename: Response to reviewers PONE.docx [file pone.0287020.s002.docx]

**Response to reviewers PONE-D-22-20005**

**Editor**

| **Comment** | **Response** | **Changes made to MS** |
| --- | --- | --- |
| Please ensure that your manuscript meets PLOS ONE's style requirements, including those for file naming. The PLOS ONE style templates can be found at  <https://journals.plos.org/plosone/s/file?id=wjVg/PLOSOne_formatting_sample_main_body.pdf> and  <https://journals.plos.org/plosone/s/file?id=ba62/PLOSOne_formatting_sample_title_authors_affiliations.pdf> |  | Done |
| Please include your tables as part of your main manuscript and remove the individual files. Please note that supplementary tables (should remain/ be uploaded) as separate "supporting information" files. |  | Tables are now included in the main manuscript. Please note that following a reviewer comment we added our raw data as a new supplementary table. |
| Please provide an amended statement that declares *all* the funding or sources of support (whether external or internal to your organization) received during this study, as detailed online in our guide for authors at <http://journals.plos.org/plosone/s/submit-now>.  Please also include the statement “There was no additional external funding received for this study.” in your updated Funding Statement.  Please include your amended Funding Statement within your cover letter. We will change the online submission form on your behalf. |  | We included the amended funding statement included in the cover letter. |
| Thank you for stating the following financial disclosure:  This study was partly funded by the European Maritime and Fisheries Fund (EMFF) grant no. 15982000049.  Please state what role the funders took in the study.  If the funders had no role, please state: "The funders had no role in study design, data collection and analysis, decision to publish, or preparation of the manuscript." If this statement is not correct you must amend it as needed.   Please include this amended Role of Funder statement in your cover letter; we will change the online submission form on your behalf. |  | Our statement on the role of the funders in the study is now included in the cover letter. |
| Thank you for stating the following in the Acknowledgments Section of your manuscript:  This study was partly funded by the European Maritime and Fisheries Fund (EMFF) grant no. 15982000049.  However, funding information should not appear in the Acknowledgments section or other areas of your manuscript. We will only publish funding information present in the Funding Statement section of the online submission form.   Please remove any funding-related text from the manuscript and let us know how you would like to update your Funding Statement. Currently, your Funding Statement reads as follows:  This study was partly funded by the European Maritime and Fisheries Fund (EMFF) grant no. 15982000049.  Please include your amended statements within your cover letter; we will change the online submission form on your behalf. |  | We removed this funding statement from the Acknowledgements |
| We noted in your submission details that a portion of your manuscript may have been presented or published elsewhere. Please clarify whether this publication was peer-reviewed and formally published. If this work was previously peer-reviewed and published, in the cover letter please provide the reason that this work does not constitute dual publication and should be included in the current manuscript. | Indeed, our samples contain a set of samples that were used previously, in van der Reijden et al. (2017). Our submission more than doubles the number of observations, and also tries to explore new ways of minimizing unwanted mortality in fisheries. Hence, the work does not constitute double publication and the data should be included in the current manuscript. | None |
| Your ethics statement should only appear in the Methods section of your manuscript. If your ethics statement is written in any section besides the Methods, please move it to the Methods section and delete it from any other section. Please ensure that your ethics statement is included in your manuscript, as the ethics statement entered into the online submission form will not be published alongside your manuscript. |  | We moved the Ethics statement to the Methods section |
| Please include captions for your Supporting Information files at the end of your manuscript, and update any in-text citations to match accordingly. Please see our Supporting Information guidelines for more information: <http://journals.plos.org/plosone/s/supporting-information>. |  | Done |

**Reviewer 1**

| **Comment** | **Response** | **Changes made to the MS** |
| --- | --- | --- |
| *Main concerns* |  |  |
| 1. Uncertainty estimates, i.e. 95% CIs, for the 12% (dry) and 16% (water-filled) should be added. The effect of water-filled hopper is only marginally significant in the final model (p = 0.027), so the emphasis on this result feels overstated. | We agree with the reviewer.  Note that we re-analysed the data following comment nr.3 (employing only the hauls for which we have paired samples of dry and water filled hopper to test for hopper effect) the hopper effect is no longer significant. | 95% CIs, for the 12% overall survival probability (dry hopper) were added were added.  Several changes were made to the MS shifting emphasis from the no longer sign increase in survival to the limited effect of the hopper. |
| 2. The result also seems overstated because I would characterize both 88% and 84% mortality as high—and the true mortality of releasing fish at sea may (probably?) be even higher. There is no discussion of how survival in a lab tank after being caught differs from surviving being thrown back into the sea. My guess is that lab survival would be an overestimate, but I can also imagine reasons why survival back in the sea could be higher. I think at least acknowledging this uncertainty in your discussion is warranted. | We agree with the reviewer. | A paragraph on how captive observation may affect observed survival has been added to the Discussion. This section also discusses the effect of the low survival among control fish in some trips. |
| 3. Many of the trips with low control survival used dry hoppers but not water-filled hoppers, and it is not clear which data were used in the GLMM analyses. Sorry if I should see this, but it is not clear to me after multiple readings. At first, I assumed the control fish were included and represented by the intercept, which would inform the model about the haul/trip random effects vs. the dry hopper effect for hauls without water-filled hopper data. But I don’t see a “Hopper: Dry” effect in Tables 4 and 6. Were all the dry and water-filled hopper data included, but not the control? This seems important because there are several hauls with low survival of control and dry hopper fish that did not have water-filled hopper fish.  I think the most proper handling of these data would be to only include hauls with both dry and water-filled hopper fish in the GLMMs. This seems more in line with how the experiment is portrayed in the methods, twin trawls with one cod end going into each hopper type, a paired design to test the effect of hopper type. An alternative could be to remove hauls with low, e.g. 50%, survival of control fish. I understand the random effects of trip and haul may somewhat mitigate this, but it doesn’t seem sufficient. | We agree with the reviewer that the most proper way to test for a hopper effect is to employ only the subset of hauls with paired observations of dry and water-filled hopper fish in the GLMMs. The hopper effect is then no longer significant.  Data of controls were not included in the GLMM analyses. Analysis of hopper effects employed only the paired observations of dry and water filled hoppers. | We made the description of the statistical methods and the data employed to test for hopper effect more clear in M&M. |
| 4. There is no data availability statement and raw data are not included in the article. | We agree with the reviewer that we should include the raw data. | Data are now available as supplementary file. (NOG DOEN) |
| *Line specific comments* |  |  |
| 25 uncertainty estimates should be provided for the 12% and 16% effects, especially as they are the main results | We agree with the reviewer. Note that the hopper effect was not longer significant and that we therefore removed the survival for the water filled hopper from the abstract | 95% CI was added for the overall survival. |
| 36 would be useful to have rough numbers instead of “part” | Unfortunately it is not straight forward to quantify the effect of temperature on survival in the studies we refer to. E.g. Van Der Reijden et al compares low and higher temperature ranges using hazard ratios. To have rough numbers we’d need to elaborate on the detailed differences between these studies, which we feel is redundant because we just want to point out here that temperature can affect survival. | None. |
| 96 should this be 117 total hauls (27 + 45 + 45)? | Thanks for noting this inconsistency. We checked the raw data and noted that the number of hauls with paired samples for the hopper treatments was 46 and not 45. The total # of hauls is thus 27+46+46 = 119.  46 ipv 45 | 45 changed to 46 |
| 189-229 please clarify which data were used in the GLMM analyses (long comment #3 above) | We agreed with the reviewer that this needs more clarification. | We added text to clarify this point. |
| 277 calling this the “final GLMM” is confusing because you have one more model below | Agree | ‘final GLMM’ was changed into ‘GLMM” |
| 301-307 this model is not described in the methods or here. Since it is a subset of the model on line 216, and similar to 283, perhaps describing how it differs is enough (i.e. exchange ‘hopper type’ for ‘vitality’, and no interaction). Clarify here that random effects of haul and trip were included. | Agree | As suggested by the reviewer the differences between this model and the model mentioned in line 216 (line 237 in the revised MS) are now described, including a clarification on the random effects. |
| 307 again, uncertainty estimates should be provided for the 12% and 16% effects | Agree. Note that we changed the text because the water filled hopper effect is no longer significant. | 95% CI, for the 12% (dry) was added. |
| 394-401 I don’t think it is fair to say “The limited effect of the water-filled hopper can be explained” or “it is likely that the contribution… increases” here. This paragraph lays out a reasonable hypothesis, but this wording seems too strong. Suggest “could be explained” on 394 and “it is possible” on 401. | Since the effect of the water filled hopper is no longer significant we rewrote this entire section. We point out that the water filled hopper results in slightly more vital fish that does not result in a higher survival. Note that this does not really change our conclusions on the effect of a water filled hopper: we state that a water filled hopper (or any other post capture measure) that does not undo damage done to the fish in the trawl can only have a limited effect on survival. | Various changes to the text because the hopper effect is now no longer significant. Please refer to the track-changes version of the MS. |
| Tables 5-6 please include the random effect standard deviation estimates + SE, these are fixed effects in the GLMMs too. | We agree that the random effects for the model are relevant. However, decided to include these in the main text of the manuscript. they are thus available to the reader | None |
| Table 5 why was the control survival so low in Trips 3, 4, 6, and 11 (12-31%, whereas most in 2017-2018 are 72-100%)? And why were none placed in the water-filled hopper? Could add to discussion. | The exact causes for the low survival among control fish in these trips are not known. However, since most control fish died in the second monitoring week while most test fish died in the first week, we suspect that causes for mortality are different. Mortality among test fish is believed to be mostly fisheries induced while mortality among control fish is mostly related to experimental procedures and conditions.  Control fish were not placed in hoppers (either dry or water-filled) because it would be impossible to detect them within the rest of the catch for sampling from the sorting belt. | A section on low survival and the effect of captive observation on the results has been added to the Discussion (same section as added for comment no. 3). |

**Reviewer 2**

| **Comment** | **Response** | **Changes to the MS** |
| --- | --- | --- |
| The authors use a title which indicates that they think the difference between 12 and 16% survival is “moderately” high. Well, 16>12, but the difference is rather low. Would another study really show the same difference (reproducibility)? Therefore, isn´t the more appropriate (and interesting) message rather the other way around: surprisingly little effect of water-filled hopper…?! | We agree with the reviewer. Note that after re-analysis of the data following a comment of reviewer 1, the hopper effect on survival is no longer significant. This of course required a change of the title.  Note that in hindsight we find it not so surprising that the water filled hopper has little (on vitality) to no effect (on survival) as it does not undo fatal damage done to the fish in the trawl. In our opinion this provides the insight that measures aimed at increasing discards survival need to focus on the trawling process because most damage to the fish seems to be done in the trawl rather than in catch processing on board. | We changed the title to **Effects of environmental conditions and catch processing on survival probability of plaice discards in the North Sea pulse trawl fishery**  The hopper effect on survival not being significant anymore led of course to various other changes in the MS. Please refer to the track-changes version. |
| Abstract: 1 or 2 introductory sentences into the research topic/question would be welcome. This should be taken up in the last sentence of the abstract.  Conditions at sea: quite unspecific – why not mention (some key) metrics considered? | We agreed with both comments. | We added the following introductory sentence:  “Undersized European plaice dominate the discarded fraction of the catch of the beam trawl fisheries for sole in the Southern North Sea.”  Re. “conditions at sea” we added “such as wave height and water temperature” |
| L 15: … undersized European plaice discarded… The fish were not discarded but selected, transported etc and kept in tanks. The wording is suggestive. | We agree with the reviewer that the fish of which survival was measured were not discarded but sampled (randomly, not selected), which is stated in the following sentence.  To make this more clear we added “from the sorting belt” .  This also makes clear that fish were not sampled from the hoppers themselves. | “Undersized plaice were sampled from both hoppers.”  Was changed into:  “For both hoppers, undersized plaice were sampled from the sorting belt.” |
| L 16: 15 trips: the fact that these trips were carried out during 4 different years is a major shortcoming of the study. It is not clear why pulse beam trawlers were used. | We respectfully disagree and in our opinion the data collection during multiple years contributes to the coverage of the variable conditions under which fish are discarded and thereby the representativity of the results.  Meanwhile in the Introduction we now explain why plaice and pulse trawling were studied. | To make clear why pulse beam trawlers were used we added this sentence to the introduction:  “The mesh size of 80 mm used in the Dutch beam trawl fishery in the Southern North Sea, combined with a minimum conservation reference size of 27 cm for European plaice, results in this species dominating the discarded fraction of the catch.” |
| L 18: fish condition: could be misunderstood e.g. with Fulton´s K (fish body condition). | We agree and since we changed condition into vitality status (as this is a more accurate description of what we measured), there is no longer a risk of misunderstanding. | Fish condition changed into vitality status. |
| L 25: is the difference between 12 and 16% significant? If on average 88 or 84% of the undersized plaice die, shouldn´t measures others than changing to a water-filled hopper be considered? It seems to be taken for granted that so many fish have to die. Maybe the fishery has to change, irrespective of whether a water-filled hopper can reduce mortality by 4%...? If the conclusion really to recommend water-filled hoppers and maybe not, to critically re-consider the way this fishery is conducted and think if there are no better measures to reduce the discard (e.g. by gear modifications)? For me the most important/robust findings of this study are only “hopper has no effect”, and the usefulness of the vitality class (published in van der Reijden et al 2017. already). | We agree with the reviewer than this increase is not ‘practically relevant’ and we do not recommended the use of water-filled hoppers to reduce discard mortality. Instead we argue that to increase survival attention should shift from the on board catch processing to the trawling because this is where the damage seems to be done.  Note that re-analysis of the data following a comment by Reviewer 1 resulted in the hopper effect on survival being no longer significant. This of course led to various changes throughout the MS. However, this does not change our original argument that to increase survival attention should shift from the on board catch processing to the trawling because this is where the damage seems to be done. | The hopper effect on survival not being significant anymore led of course to various other changes in the MS. Please refer to the track-changes version. |
| Keyword: discard survival; “abiotic variables” doesn´t seem to be a useful term here – anything more specific? Or instead North Sea or something related to the method? Pleuronectes platessa? | Agree | ‘abiotic variables’ replaced by ‘North sea’ |
| Several of the issues mentioned in this review were not appropriately touched upon in this Introduction, e.g. large variability in discard studies, correlational vs causal relations, multi-species catches but focus on plaice (why only one species? Why plaice?), why pulse beam trawlers, why should a water-filled hopper be the solution to the high discard mortalities?…) | We largely agree with the reviewer that certain issues are not appropriately touched upon in the Introduction.  **Large variability in discards studies** (interpreted as large variations in observed discards mortality): is mentioned in Line 40.  **Why only one species? Why plaice?:**  Our research trips assessed discards survival for several species next to plaice (sole, turbot, brill, thornback ray) but with lower numbers of observations. This paper reports only on plaice because the paper focusses on the effects of conditions at sea and water-filled hoppers on discards survival. Only for plaice the number of observations was sufficient to assess these effects and only plaice was sampled from both the dry and water-filled hopper.  Re. the water-filled hopper we opted for a larger number of observations on a single species rather than dilute the number of observations (limited by capacity of the holding units) by sampling multiple species. Plaice was chosen because it dominates the discarded bycatch and appears in sufficient numbers in the catches to enable balanced sampling. We consider plaice as an indicator species for the hopper effect: if plaice benefits from a water-filled hopper we consider it likely that other species will benefit as well. This is appropriate for this ‘proof of principle’ approach towards the potential effect of a water-filled hopper.  **Why pulse beam trawlers?**  Because at the time the experiments were conducted this was the dominant Dutch demersal fisheries in the North sea.  **Why should a water-filled hopper be the solution?**  A previous study suggested promising positive effects of a water-filled hopper (mention in line 47-49). This combined with the water-filled hopper being a measure that could be relatively easy implemented in the current fishing and fish processing methods, makes it a logical one of the first measures to explore. | **Large variability in discards studies:** no changes to MS  **Why only one species? Why plaice? Why pulse beam trawlers?**  We added the following sentence to the Introduction:  “The mesh size of 80 mm used in the Dutch beam trawl fishery in the Southern North Sea, combined with a minimum conservation reference size of 27 cm for European plaice, results in this species dominating the discarded fraction of the catch.”  **Why should a water-filled hopper be the solution?**  The fact that a water-filled hopper could be relatively easily implemented is now mentioned in the Introduction. |
| It seems that the factors Vessel, Area, and Year were not accounted for in the analysis. And apparently there was no pairwise testing of vessel-trip on the treatment hopper. | The effect of vessel, area, and year are not explicitly included in the model, but are accounted for by adding the random effect for haul, or haul and trip.  Meanwhile, in the revised MS, the testing of the hopper treatment was changed so that the test for this effect now only includes trips where both hoppers were present | The testing of the hopper type was changed, also in response to the other reviewer. The results changed as a result: while the effect size of the hopper type on survival is small, as previously, the effect is not significant in the updated results. |
| L 77: In the study 15 trips on 4 commercial trawlers were used. The factor vessel (inter-vessel variation) is not accounted for in the analysis despite the fact that vessel (closely related to the specific gear and the performance of the captain with his ship on a particular fishing ground) is often a major source of variation. | While indeed there is no vessel effect, there is a random effect of trip and haul. These “absorp” any effect of vessel, because trip and haul are unique to a vessel. Hence, any differences between vessels is now included in the effects of trip and haul. | None |
| The sampling covered the time period 2014-2018, with no sampling in 2016, i.e. 15 hauls from 4 trawlers from 4 years. However, there are no samples from April or August. | Correct, see also 2nd comment below  Seasonal variation was captured by the temperature range | None |
| The factor year is not accounted for the analysis (inter-year variation). Moreover, different year classes of plaice must have been caught in different years. Stronger or weaker year classes may bring fish of different fitness/condition factor; this was not accounted for in the analysis. | This certainly is an interesting hypothesis. Unfortunately we have no body mass data to calculate condition factors and thus cannot assess the effect of differences in condition (if any) on survival. | None |
| It seems that the sampling did not follow a strict temporal design, so that many outside (environmental) factors could not be controlled and are assumed to be accounted for by the more-or-less randomness of how the samples were taken. | The reviewer is correct and in our opinion, this is inherent to the nature of this kind of field research in collaboration with commercial fisheries. Because conditions at sea cannot be controlled, a balanced experimental design with respect to variables like wave heights, wind speeds etc. is very hard if not impossible to achieve. The best that can be done (and was done) is to spread out trips over the year to cover as much variation in conditions at sea as possible within the logistical limitations that are inherent to conducting research with commercial fisheries. | We now elaborate on this in the Discussion. (Line 388-394) |
| L 89: How did the authors ensure that a single fish could be backtracked to a specific haul and hopper when the fish were kept in tanks prior to tagging? | This was ensured as follows:  - fish were tagged and housed in tanks after a sampling of a haul; untagged fish from different hauls could never be mixed.  - for hauls with hopper treatment, the two hoppers were processed separately (in consecutive order) allowing to sample fish for each hopper from the sorting belt.  - fish sampled from each hopper were put in separate labelled tanks prior to tagging. | To clarify this the following sentence was added to the section on Assessment of fish condition and monitoring of survival:  “To ensure backtracking of each test-fish to its haul and hopper treatment, test-fish from different hopper treatments were stored in separate 105L holding containers prior to tagging. Tagging was completed before the next haul was sampled.” |
| L 90: Seven out of 15 trips had… | Agree | Changed as suggested by reviewer |
| L 90: For each of the 15 trips, test-fish… Unclear how the fish were selected. For instance, please clarify if each time a balanced number of fish was selected. How did you chose when to pick which fish? | We agree with the reviewer that the description is incomplete and as a result unclear. All fish were randomly picked from the sorting belt (no selection). Each haul fish were taken at the start and end of the catch sorting process in equal numbers (5 to 10).  For the hauls with a hopper treatment we picked the first 5 and last 5 from each hopper, resulting in a sample of 10 per hopper treatment and a total of 20 fish per haul to process. | The originally last part describing the catch sorting process was moved up to become the first part of this section. We believe it is more clear to mention this generic information before the specific information for the subsets of trips.  We now mention that test-fish were *randomly* sampled.  We added the description of sampling for hauls with no hopper treatment. |
| L 96: … 27 hauls. During the other 8 trips (and a total of 20 hauls), both a … were used, and 10 fish per … | Agree | Changed as suggested by reviewer |
| I couldn´t find information on the duration of the hauls (range, mean, SD), and if the fishery was conducted at day and/or night or 24 hs. Time of day can influence the catch composition and hence the survival probability. It is unclear what the range and mean of the total catch weight per haul was. The species composition of the hauls is not given and was not accounted for in the analysis (e.g. round fish (with gas bladders) can make the cod end float; flounder or other organisms with a rough surface can hurt the skin and mucosa of plaice). | Haul duration (range) is mentioned in Table 3.  The fisheries was conducted around the clock (24 h/day). However the test-fish were not sampled at night.  We agree with the reviewer that catch weight and composition may affect discards survival probability. Unfortunately we have no reliable data on total catch weight per haul and no data on catch composition to include in the analysis. Accurate total catch weight is notoriously difficult to determine. Skippers did record their estimates of total catch weight for each sampled haul but we decided that these estimates were too unreliable to include in the data analysis. Total marketable fish was also recorded but because of large variation in amounts of discards (visual observation), total marketable fish is a poor indicator for total catch weight.  We consider variation in total catch weight and catch composition as ‘natural’ variation of the fisheries which was accounted for in our data by sampling test-fish from many hauls year-round at different fishing grounds. | None |
| L 94: where do the 45 hols come from? | This is the sum of the number of hauls for trip 7 -10 and trip 12 -15 during which 2 hopper treatments were employed. Note that we made a mistake and that the total number of hauls for which data on hopper treatments was collected was actually 46 and not 45. See Also Table 2. | 45 was changed into 46 |
| L 96: Please clarify: x+y+z = 119 What is x,y,z? Reference to Table 2 at the end of the paragraph could be useful. | 119 is the total number of observed (group) discards survival probabilities.  27 hauls for dry hopper only, 46 (not 45) hauls for dry & waterfilled hopper results in 27 + 46 + 46 = 119 observed (group) discards survival probabilities. | A reference to Table 2 was added. |
| L 97: ... were sampled onboard during … | Agree | Changed as suggested by reviewer |
| L 106: assess instead of separate? | Agree | Changed as suggested by reviewer |
| L 111: fish were fed once (?) daily with… At what time? | Agree. Fish were fed in the mornings. | Changed to .... fish were fed *once* daily *in the morning* with ... |
| L 126: Substrate type for each haul was … | Agree | Changed as suggested by reviewer |
| L 129: used for survival – syntax unclear | Agree | changed to:  ...linked to individual hauls used for *collecting test-fish*, based... |
| L 134: in 105L plastic (?) holding … | Yes | ‘plastic’ was added |
| L 137: Tagging, especially in smaller fish, could have caused an additional source of mortality and an additional source of variation (if e.g. tolerance against tagging differs between seasons) which was not assessed in the experiments. | We agree with the reviewer that tagging poses additional stress and injury but we consider that effects on our results are unlikely. When dead fish were detected and removed from their tank, we immediately dissected the tags from their body. Although the objective was to recover the tag and discarding carcasses without tags, this provided the opportunity to observe the tagged area of all fish that died in the experiments. Excessive internal haemorrhages around the tag were observed in a few cases (none of which were control fish). In these cases tagging may have accelerated mortality but given the overall condition of these fish, tagging was never considered the main cause of death (most likely the fish would have died anyway) and we therefore did not remove these fish from the data. | None |
| L 138: Fish condition could also be understood as fish body condition, such as Fulton´s condition factor. Maybe use more unambiguous terminology | We agree with the reviewer, also because of harmonization with the terminology used in scientific literature. | Fish condition was changed to ‘vitality status’ or ‘vitality’ (depending on the context) throughout the entire manuscript. |
| L 139: Were different persons involved in the assessment? How was their performance compared between each other? And was the factor “person/assessor” accounted for in the analysis? | Yes different persons were involved in data collection at sea. The method for vitality assessment was standardized in a written protocol and by joint training of the persons scoring the fish prior to data collection at sea.  Vitality assessments always involved 2 persons (one handling the fish, one writing the scores). The scoring of external injuries and reflex impairments enforced a systematic assessment of each fish, resulting in a summarizing vitality class (A to D) that was used in data analysis.  Jointly taken, we employed procedures to minimize observer bias, we believe observer bias is minimal but cannot entirely exclude it. Performance of observers was compared nor accounted for in the data analysis. | We expanded the text to clarify the number of observers and their training.  The text now includes:  “jointly by two observers”  “Vitality assessment was standardized in a protocol and by joint training of the observers. “ |
| L ... The experimental facilities deprived the fish from any natural predation (e.g. sea gulls at the water surface, and any other aquatic vertebrate or invertebrate predators in the sea), from parasites and bacteria, viruses and other species interactions that a flatfish would encounter at the sea floor post-discard and which all are likely to influence and further increase the discard mortality. But the artificial monitoring units used in this study could also lead to unnatural mortality because this may also have caused additional stress. Hence the results are obviously not directly transferrable to conditions in the wild and are biased. How relevant are the results for real life? One could even say that this is just an experiment and that the results are not transferable to real life. However, a discussion of these shortcomings is presently missing in the ms. | We agree with the reviewer regarding the short comings of measuring discards survival by captive observations.  At the same time, we consider this ‘the best we can do’ to assess discards survival probability (in line with ICES WGMEDS).  We agree with the reviewer that this should be touched upon in the MS and added a paragraph to the Discussion. | A paragraph on the shortcomings of captive observation to measure discards survival probabilities has now been added to the Discussion. |
| L 168: .. with dead or alive polychaetes? | Live | Inserted ‘live’ |
| L 200: The model does not account for several possible effects such as vessel, fishing ground, year or presence/absence of other species in the catches or catch composition. To me it is not fully clear how data of 8 trips with both hopper types (identical sea conditions) were analysed in comparison to the 7 trips with a conventional dry hopper. | The effect of vessel, area, and year are not explicitly included in the model, but are accounted for by adding the random effect for haul, or haul and trip.  In addition, the effect of fishing ground is partly included by having a substrate effect.  Meanwhile, in the revised MS, the testing of the hopper treatment was changed so that the test for this effect now only includes trips where both hoppers were present | The testing of the hopper type was changed, also in response to the other reviewer. The results changed as a result: while the effect size of the hopper type on survival is small, as previously, the effect is not significant in the updated results. |
| L 211: and within trips, the model … | Agree | Changed as suggested by reviewer |
| L 212: Is SST considered equal to bottom water temperature, i.e. the habitat where plaice usually live? | No not necessarily. A thermocline may exist with lower water temperatures at the bottom during periods of calm weather while storm conditions will causes mixing and temperature to equalize in the water column of the relatively shallow North sea.  We chose to use surface water temperature as in contrast to temperature at the seafloor, we could easily collect reliable data. Because SST most likely correlates well with temperature on the seafloor, it is appropriate to use SST in de analysis even though it may not be always identical to bottom water temperature. | None |
| L 222/223: Here the authors state that the design contains so many possible interactions… | Indeed we tested for all one-way interactions. However, the final model found in our model selection procedure did not contain any interactions. | None |
| L 229: is fiducial the right term? Significance level was set at 5%. | Fiducial is not incorrect but signifcance level is more common to use. | Changed to significance level |
| **Results** |  |  |
| L 242: revealed… was relatively high. The use of the term “test-fish” and “control fish” could be confusing. | Agree, also the reference to Fig 1b (survival by vitality class) is not correct as this does not include control fish. | Corrected and made more clear. |
| L 248: Mean survival of the fish : of which fish? Test.fish, or control fish or plaice? In Fig. 1 I see a continuous decline for control vs a drop at the beginning and plateau after ~7 days for the other fish | Mean survival of test-fish. | We changed ‘fish’ to ‘test-fish’ to avoid this confusion. |
| L 273: Finally, the standard … | Agree | Changed as suggested by reviewer |
| L 274: and was does this mean then?. | This means that the standard deviation of the distribution of the random effect of haul (on the intercept of the model) was 0.42. The mean of that random distribution is zero. The mean and SD combined provide a distribution of the effect of haul in the entire dataset. For more information see e.g. Zuur et al. 2009 “Mixed Effects Models and Extensions in Ecology with R” | Finally The standard deviation for the distribution of the random intercept effect of haul was estimated to be 0.42. |
| L 278, 279: maybe consider to use SST instead of temperature | Agree | Changed to ‘surface water temperature’ |
| L 294: temperature-dependent | Agree | Changed as suggested by reviewer |
| L 295: … was only 12% | We agree with the reviewer that the survival is low and *only* 12%. However, we prefer to avoid subjective statements in de Results section and therefore not to change this as suggested by the reviewer. | None |
| L 296: negligible: please say how high or low this was. | We changed the text to describe the actual percentage | For vitality class D survival at 4 °C was 12% ( 95% CI: 51%-69%), decreasing to a negligible survival at 12 °C. Was changed to “For vitality class D survival at 4 °C was 12% ( 95% CI: 4%-29%), decreasing to a survival probability of less than 0.1% at 12 °C.”. Note that this sentence also corrected a typo in the 95% CIs for the 4 degrees C estimate of survival. |
| L 299: …/(class C), it was linked to an increase in survival from 1% to 7% This is still very low an mean that 99 alias 93% died! | We agree with the reviewer that the survival is still very low.  At the same time, length has a marked effect on survival probability that should be mentioned (a seven fold increase from the smallest to the largest fish in the data).  We just objectively state our results. | None |
| L 301-303: wording unclear to me. Vitality class was depended on hopper type? Was there a bias in the fish that came from the different hopper types? | Indeed vitality class depended on hopper type: vitality of fish from the water-filled hopper was slightly better than of fish from the dry hopper. This is not bias but a treatment effect in our opinion. | The paragraph was revised to make it more clear. |
| L 303, 304: showed, not shows | Agree | Changed to ‘showed’. |
| L 306: how useful / relevant is a mean temperature? | Note that this phrasing is inherent to the model approach: single factor effects are quantified by fixed the other significant variables at their mean values.  Note that we changed the text following the hopper effect being no longer significant. “mean temperature” is no longer mentioned. | The text was changed because the hopper effect is not significant, making the mentioning of “mean temperature” redundant. |
| L 30/: or mortality decreased from 88 to 84%. What is the confidence interval around these estimates? Given the number of factors potentially playing a role in influencing survival, is this really considered a difference that is stable and reproducible and worthwhile to be called a difference? Maybe the message is rather the other way around: surprisingly little effect of hopper type and an overall high mortality of experimental plaice. The whole aspect of the use of alternative fishing gears that can reduce the bycatch before it is hauled onboard is not discussed at all. Certainly, fishing gear which avoids the capture of non-targeted fish sizes under water and during the capture process are much more likely to reduce bycatch and hence survival of fish than hopper type. This may have to be mentioned in the discussion. Presently, the reflections on alternative measures are rather shallow. | We totally agree with the reviewer that the effect of the water-filled hopper is very small if not absent and that it is certainly not the solution for the high discards mortality in this fisheries.  We already mentioned in the Discussion that a water-filled hopper has no effect on survival because the damage to the fish is already done before it arrives on deck, and that therefore further research should focus on the impact of the catch and hauling on the fish. | Changes were made because hopper effect was no longer significant.  We now added a statement that the water-filled hopper is not a solution for the high mortality among plaice discards.  We also added that further research should also focus on avoiding catching undersized plaice |
| **Discussion** |  |  |
| L 32=: with an annual SST range of 6 to 17 °C | Agree | Changed as suggested by reviewer |
| L 323: and higher mortalities observed in summer (temperature range: x-y °C) – and delete next sentence | Agree | Changed as suggested by reviewer |
| L 327: do the fish experience hypoxia on deck? They rather suffocate because their gills don’t work outside water. Hypoxia operates when the O2 level of the water is very low, but when the fish are out of the water, hypoxia can´t apply. The reasons for higher survival in winter may be more complex and may involve factors such as physiological/hormonal state of the fish (poikilotherms), the characteristics of the mucosa, reduced metabolism, less irradiation on deck, lower temperature difference between sea floor and deck etc. Again, many (coinciding) factors are involved. This study can only provide correlational evidence, no causal evidence. This should be acknowledged in the scientific argumentation and in the wording. | The reviewer is correct that gills don’t work outside the water. This is caused by a reduced gas-exchange area due to the collapse of gill filaments. As a result oxygen uptake is reduced and this may lead to oxygen shortage at tissue level, which is defined as hypoxia. So yes, fish can experience hypoxia on deck.  We agree with the reviewer that the reasons for the higher survival in winter may be more complex and caused by multiple factors of which we mention 2: temperature shock and hypoxia. | We added a sentence (Line 393) stating that this type of research can only provide correlation evidence and no causal evidence. |
| L 329: condition improved when using a water-filled hopper – can this really be 100% attributed to the hopper type? Was it significant? Did any extreme data drive the difference (e.g. a few hauls with very high or low survival in either of the hopper types)? The difference is really small. | While the effect was significant in the original submisison, the new approach to our analyses resulted in this effect being not-siginificant. The text was changed accordingly. Having said that, the estimated effect size did not change substantially. | The text about the significance of the hopper type was changed throughout the MS, given that new approach to our testing suggests the effect was not significant. |
| L 333: moderate? 4 % is rather minor, Or: a slight increase. I think if the authors want to follow their line of argumentation that they think that 4% is “moderate”, a table is needed showing that the difference was really consistent. My feeling is that the message is rather: hopper type has a surprisingly small effect. And then it would be really interesting to read/see how the authors argue for an “almost no-effect” outcome because their “ideological pair of glasses” prior to this study most likely was that hopper type must have an effect. The densities in the hopper are usually very high, and the fish likely have difficulties to ventilate the operculum when so many other fish are around, lots of mucus is released and the fish may suffocate anyway, not matter if water is supplied. | We agree with the reviewer that the effect was very small (and of no practical relevance) and that minor would be a more appropriate phrasing than moderate.  Re-analysis of the data following a comment by reviewer 1 resulted in the hopper effect being no longer significant. We consequently rephrased this part of the discussion. | Changes were made because hopper effect was no longer significant. |
| At no point in the Discussion do the authors explain why the study was conducted on pulse beam trawlers and not on standard beam trawlers. What was the particular benefit or reason for using a pulse beam trawler to assess the role of a hopper effect? | This study was conducted with pulse beam trawlers as at that time this was the dominant type of beam trawling by the Dutch fleet. | The reason for conducting this study with pulse trawlers has been added to the Introduction. |
| L 336: … lower water temperature… | Agree | Changed as suggested by reviewer |
| L 338: … higher temperatures… | Agree | Changed as suggested by reviewer |
| L 350: did catches from sandy grounds have more roundfish? Their swimbladders may cause the cod end to float, see e.g. Kraak et al. 2018. Or was there a vessel effect? Any additional helpful information coming from the data themselves? | We did not assess catch compositions so we have no data on differences in catch compositions between substrate types. However, round fish catches are generally low in this fisheries and floating cod ends due to swim bladders seem highly unlikely to us. | None |
| L 365: Could the paragraph be shortened? It mainly contains speculations but does little to advance our understanding about underlaying mechanisms for discard mortality. For me, this paragraph is a nice example that such studies can only provide some superficial correlational evidence, but they lack the power to understand underlaying/causal mechanisms, because 1) there are so many factors involved, 2) the fate of individuals is not considered as the focus is on integrated group means and averages. | While we agree with the reviewer that these studies can only provide correlation evidence, we think that it is important to give possible reasons why we did not find effects of covariates on fish survival that have been commonly found in other studies. Counting six sentences we find the paragraph not excessively long as it stands. | None |
| L 373: … fishing reflected commercial conditions Or settings. | This needed to be rephrased because it was inaccurate. | because *research* was done under commercial fishing circumstances. |
| L 376: … study were… | Agree | Changed as suggested by reviewer |
| L 382: … be higher, their ….survival was…catches was… | Agree | Changed as suggested by reviewer |
