# Supplementary material for: Effects of environmental conditions and catch processing on survival probability of plaice discards in the North Sea pulse trawl fishery
Source: PLoS One. 2023 Jun 14;18(6):e0287020. doi: 10.1371/journal.pone.0287020 (PMC10266594; doi:10.1371/journal.pone.0287020)
Supplement: Attachment — Submitted filename: Response to reviewers PONE R1.docx [file pone.0287020.s003.docx]

Response to reviewers PONE-D-22-20005

| Comment | Response | Changes made to MS |
| --- | --- | --- |
| 22-23 this sounds like you had both dry and water-filled hoppers on 15 trips, but there were only 8 trips with water-filled hoppers. Could change to ‘8’, since this is the sample size used in the GLMM, or perhaps remove the number, just say ‘During trips with …’ | Agreed | Changed text to “During trips with" |
| 35 cut “fish” | Agreed | Changed text, removing “fish” |
| 65 is “high survival” defined with a number/range? If so, please include. In either case, it would be nice if you can clarify what, specifically, fisheries need to document to obtain an exemption. | Agreed | Text has been changed to explain that “high survival” has not been defined to date. |
| 91 Trips | Agreed | Changed text from “Trip” to “Trips” |
| 285-286 suggest ‘where both hopper types were used in a paired design.’ | Agreed | Added “where both hopper types were used in a paired design” |
| 528-529 The water-filled hopper effect was pretty close to significant, p = 0.057, so I suggest changing this and adding the estimate of survival in the water-filled hopper treatment. Something like, ‘Overall, the estimate for the survival probability for plaice discarded by pulse trawl fisheries was low (12% with 95% CI: 8% - 18%). The water-filled hopper had a weak positive effect on survival (p = 0.057, 16% with 95% CI xx%-yy%).’ And then a final sentence with your recommendation from previous paragraph, a summary of ‘We therefore recommend that future studies into increasing discards survival probabilities focus on avoiding unwanted bycatches of undersized plaice, i.e., improve the size selectivity of the gear and on increasing the proportion of fish in good condition in the catches by reducing the impact of capture and hauling processes on fish condition.’ | Partly agree. | While we agree that the effect of the water filled hopper is close to significant, we would like to keep a strict formulation of our results , when these are “not significant” (if p >0.05). Hence, we keep the text as it is.  Those readers who would like estimate the survival in the water-filled hopper, can easily calculate the mean survival rate from Table 6.  Meanwhile, we did finish the conclusion section with a summary of our recommendation, as per the suggestion of the reviewer. |

=d
